# Removing Random Noise of GPR Data Using Joint BM3D−IAM Filtering

**DOI:** 10.3390/s25103246

**Published:** 2025-05-21

**Authors:** Wentian Wang, Wei Du, Zhuo Jia

**Affiliations:** 1 Institute of Disaster Prevention, Sanhe 065201, China; wwt@cidp.edu.cn; 2 Hebei Key Laboratory of Earthquake Dynamics, Sanhe 065201, China; 3 Langfang Key Laboratory of Earth Exploration and Information Technology, Sanhe 065201, China; 4 College of Geo-Exploration Science and Technology, Jilin University, Changchun 130021, China; duwei@ynu.edu.cn; 5 School of Earth Science, Yunnan University, Chenggong Campus, Kunming 650091, China; 6 School of Civil and Environmental Engineering, Changsha University of Science & Technology, Changsha 410114, China

**Keywords:** BM3D, improved adaptive median filtering, GPR, denoising

## Abstract

Random noise degrades the quality and reduces the interpretability of Ground Penetrating Radar (GPR) data. The Block Matching Three Dimension (BM3D) algorithm is effective at suppressing Gaussian noise, but ineffective at handling salt-and-pepper noise. On the other hand, the Improved Adaptive Median (IAM) filter is suitable for eliminating salt-and-pepper noise, but performs poorly against Gaussian noise. In this paper, we introduce and implement JBI, a joint denoising algorithm that integrates both BM3D and improved adaptive median filtering, exploiting the advantages of both algorithms to effectively remove both Gaussian and salt-and-pepper noise from GPR data. Applying the proposed joint filter to both synthetic and real field GPR data, infested with various proportions of different noise types, shows that the proposed joint denoising algorithm yields significantly better results than these two filters when used separately, and better than other commonly used denoising filters.

## 1. Introduction

Ground Penetrating Radar (GPR) is an effective geophysical method for detecting subsurface metallic and non-metallic objects by transmitting and receiving high-frequency electromagnetic waves [1,2,3,4,5,6]. GPR primarily relies on the differences in relative permittivity and conductivity between the subsurface object and the surrounding medium for distinction [7,8,9,10]. GPR is widely used in various fields, including but not limited to geological, hydrogeological, engineering, environmental, archaeological, and geotechnical investigations [11,12,13,14,15,16,17,18].

GPR data usually suffer from noise interferences during data acquisition, which seriously affects the signal-to-noise ratio (SNR) of the collected data [19,20,21,22,23,24]. These noises include regular noise and random noise [25,26,27]. Among them, random noise is mainly composed of Gaussian noise and salt-and-pepper noise [28,29,30,31,32,33]. Gaussian noise mainly originates from scattering by uneven bodies, such as underground soil particles, as well as various random disturbances [33,34,35,36,37,38,39,40,41,42]. The main characteristic of Gaussian noise is its normal distribution with a mean of zero, while its variance represents the strength of the noise. Salt-and-pepper noise mainly arises from minor instrument instability, partial loss of transmitted data, or large-scale disturbances caused by other reasons [43]. Its main characteristic is having a local maxima and minima. These two types of noise are the most common forms of random noises, and GPR data are often contaminated by a mixture of both, which significantly degrades data quality.

Median filtering and mean filters are effective traditional methods for removing random noise [44]. They exhibit significant filtering effects on both Gaussian noise and salt-and-pepper noise. Median filtering excels at filtering salt-and-pepper noise. Conversely, median filtering may introduce errors during the process of replacing original values with medians, as the medians may still be contaminated with noise. Moreover, original values are occasionally not contaminated by noise, but are replaced by median filtering [45]. Such drawbacks motivate researchers to propose and implement the Improved Adaptive Median (IAM) filtering approach, which processes only noise-contaminated points, leaving non-contaminated points intact [45]. This approach guarantees that replacement is only performed if the original value is contaminated by noise, significantly enhancing the filtering effect against salt-and-pepper noise. However, the improved adaptive median filtering method is mainly effective against salt-and-pepper noise and is less effective against Gaussian noise. Given that noise in measured data often consists of a mixture of both types, it is vital to create an effective filter against both types of noise. Gaussian noise, with a mean of zero, is more easily removed by mean filtering. However, traditional mean filtering tends to smooth out image details, severely damaging detailed information from the useful signals. Therefore, in 2007, Dabov et al. [44] proposed the Block Matching Three Dimension (BM3D) algorithm. The basic concept of BM3D stems from the fact that natural images inherently contain many similar repetitive structures. It employs image block matching to collect and aggregate these similar structures, and then performs orthogonal transformation to obtain a sparse representation. Leveraging sparsity and structural similarity, BM3D performs filtering. BM3D denoising can fully preserve the structure and details of the image, achieving a good SNR. The BM3D denoising effect on Gaussian noise is significantly better compared to mean filtering. However, numerical simulation experiments have shown that the BM3D algorithm performs poorly against salt-and-pepper noise, indicating its evident limitations.

As most GPR noise is a mixture of Gaussian noise and salt-and-pepper noise, we propose applying the IAM filter, with a specific window size, to remove the salt-and-pepper noise from the GPR data. Then, we select several regions much larger than the window, calculate the variance of the data within each region as an estimate of noise variance, and then apply the BM3D algorithm to remove the Gaussian noise. This approach combines the advantages of both the BM3D and IAM filters. The next sections introduce the results of applying the proposed joint filter to both synthetic and real field GPR data. They also introduce a comparison between the results of the proposed joint filter with traditional mean, median, IAM, and BM3D filters, as well other commonly used and recently introduced filters.

## 2. Methods

Random noise is a common type of noise in GPR data. Typical random noises include Gaussian noise and salt-and-pepper noise. In practice, noise affecting GPR data is often not simply Gaussian noise or salt-and-pepper noise, but rather a superposition of the two. Therefore, in the following, the mathematical models of Gaussian noise and salt-and-pepper noise are discussed separately.

### 2.1. Mathematical Models of Common Random Noise

Gaussian noise refers to a type of noise whose probability density function follows a Gaussian distribution, with the following probability density function:

(1) $$P\left(z\right)=\frac{1}{\sqrt{2\pi }\sigma }e^{-\frac{{\left(z-\mu \right)}^{2}}{2\sigma^{2}}}$$

where the *z* represents the grayscale value of the image, *μ* represents the expectation of *z,* and *σ*^2^ represents the standard deviation of *z*. Gaussian noise is concentrated around the mean value *μ*, and the larger the standard deviation *σ*, the greater its dispersion. Specifically, noise with *z* values in the interval [*μ* − *σ*, *μ* + *σ*] accounts for approximately 68.2% of the total noise, while noise with *z* values in the interval [*μ* − *2σ*, *μ* + *2σ*] accounts for approximately 95.4% of the total noise.

Salt-and-pepper noise, also known as impulse noise, is generated when part of the data are lost due to random interference during the transmission of information recorded by the GPR instrument. Its probability density function is expressed as follows:

(2) $$p\left(a\right)=\left\{\begin{array}{c} P_{p}\;a=p\\ P_{q}\;a=q\\ 0\;others \end{array}\right.$$

where $p\left(a\right)$ is the probability density function of salt-and-pepper noise, $P_{p}$ and $P_{q}$ are non-zero and approximately equal probabilities, *p* is the maximum value, *q* is the minimum value, and a represents the data to be processed.

Salt-and-pepper noise exhibits maximum and minimum values; therefore, useful signals typically lie between these extremes. As a result, the probability that the maximum and minimum values within a window are noise is highest, while the probability that values between them are useful signals is lower. On rare occasions, exceptions may occur when the useful signal itself reaches peak and/or trough values, in which case it may be classified as noise.

Since our algorithm is based on a joint denoising approach combining BM3D and IAM filtering methods, a systematic introduction to these two algorithms is provided in the following sections.

### 2.2. BM3D Algorithm

BM3D denoising is an image denoising algorithm based on traditional methods, proposed by Dabov et al. [44] in 2007. This method currently boasts the best denoising performance among traditional methods for removing Gaussian noise.

The BM3D algorithm consists of two steps. The first step involves simple denoising through matching the original image to form a basic estimate. The second step involves more detailed denoising by combining the original image and the basic estimate, further improving the SNR.

In the first step, block matching, the algorithm searches for similar image blocks within a defined neighborhood of each block in the noisy image. To avoid the influence of noise, the blocks are transformed into 2D representations, and Euclidean distance is used to measure their similarity. Similar blocks are then stacked to form 3D arrays.

The 3D array is composed of pixels at corresponding positions in each stacked block. A hard threshold is then applied, setting components smaller than a given parameter to zero. At the same time, the number of non-zero components is counted to serve as a reference for subsequent weighting. Finally, an inverse transform is applied along the third dimension.

These blocks are placed back to their original positions after inverse transformation, and the superposition weight is counted by the number of non-zero components. Finally, the basic estimated image is obtained by dividing the stacked image by the weight of each point. At the end of this step, the image noise has been largely removed.

In the second step, collaborative filtering, the 3D arrays are transformed using 3D transformation. This transformation concentrates the signal energy into a few coefficients, while the noise is spread more evenly. A hard thresholding is applied to these coefficients to suppress the noise. Finally, the inverse 3D transformation is applied to obtain estimates of the original image blocks. These filtered blocks are then returned to their original positions. To reduce artifacts, the algorithm typically performs a weighted averaging of overlapping blocks. These two steps suppress most of the noise while restoring most of the details of the original image.

### 2.3. IAM Algorithm

The Improved Adaptive Median (IAM) filter was introduced by Wang et al. in 2025. The filter is based on median filtering and adaptive median filtering, which significantly improved the filtering performance of salt-and-pepper noise [45]. The following is a brief description of the algorithm, while detailed information can be found in [45]:

(1) Determine whether the data point is a local maximum or minimum, as salt-and-pepper noise manifests as local maxima or minima. If so, apply filtering; otherwise, no processing is performed.
(2) The filtering process begins with selecting the smallest window for processing.
(3) Divide the filter window into four sub-windows, compute the median for each of the four sub-windows, then compute the median of these four medians. If the median is neither the maximum nor the minimum, use the median to replace the original value; otherwise, expand the window.
(4) Expand the window and repeat Step (3). When the window is expanded to its maximum size and the median remains at the maximum or minimum value, select the median of the non-maximum or non-minimum points within the window to replace the original value.
(5) Traverse the entire dataset and complete the filtering process.

### 2.4. Joint BM3D−IAM (JBI) Algorithm

The Joint BM3D−IAM (JBI) filter employs a combination of both previously introduced filters to take advantage of the capabilities of both filter against different types of random noise. The joint filter works as follows:

First, select an appropriate square. If the window is too small, it may result in an insufficient denoising capability. If the window is too large, it may lead to the loss of image details. Moreover, the window size must be odd to ensure that the point to be filtered is in the center of the window. Simultaneously, determine the step size of the variable window. If the noise density is high, then a larger value should be selected for the variable window step size; if the noise density is low, then a smaller value should be selected.

Filtering window size selection is a crucial step for the success of the filtering process. The following is a description of the filtering window size. After normalization, a small area (e.g., 5 × 5 pixels) without a signal is selected. Since this area contains no useful signal, it is considered as noise. The variance of this area is calculated and can be used to assess the noise intensity, as follows:

(3) $${\bar{a}}_{i,j}=\frac{1}{25}\sum_{m=-2}^{2}\sum_{n=-2}^{2}a_{i-m,j-n}$$

(4) $$D_{ij}=\frac{1}{25}\sum_{m=-2}^{2}\sum_{n=-2}^{2}\left(a_{i-m,j-n}-\bar{a}\right)$$

where $a_{i,j}$ is the value of the *i*-th sampling point and the *j*-th sampling point, ${\bar{a}}_{i,j}$ is the average of the data in the window. $D_{i,j}$ is the variance of the data in the window. Therefore, the window size of the IAM filter is selected as:

(5) $$windowsizeIAM_{min}=\left\{\begin{array}{l} 3,\;0⩽D_{i,j}<1\\ 4,\;1⩽D_{i,j}<2\\ ...,\;....\\ n,\;n+3⩽D_{i,j}<n+4 \end{array}\right.$$

where $windowsizeIAM_{min}\times windowsizeIAM_{min}$ is the size of the IAM window. The step size of window expansion is generally 2. The window of BM3D is generally 5 times the size of the minimum window size of the IAM window. Therefore, the window length of BM3D is as follows:

$$windowsizeBM3D=5\times windowsizeIAM_{min}$$

where windowsizeBM3D×windowsizeBM3D is the size of the IAM window.

Similarly, data density can also affect the size of the window. So, the data density can be calculated using the following formula:

(6) $$D_{data}=\frac{f_{sample}}{f_{center}}$$

where *D_data_* is the data density, $f_{center}$ is the center frequency of the received signal, and $f_{sample}$ is the sampling frequency. The window size is determined by multiplying the calculation result of Formula (5) by n.

(7) $$n=\left\{\begin{array}{l} 1,\;D_{data}⩽200\\ \frac{1}{10}\cdot \left[\frac{D_{data}}{20}\right],\;D_{data}>200 \end{array}\right.$$

$\left[\cdot \right]$ indicates the rounding operation. The starting window of the improved adaptive median filtering algorithm cannot be too large, as this can damage the useful signal while filtering out the salt-and-pepper noise. The starting window refers to the minimum window in the modified adaptive median filtering window.

Second, IAM filtering is employed to remove the salt-and-pepper noise from all points within the window. A small window size is selected, and the median is checked. If the median represents a noisy sample, the window size increases gradually until the median does not represent a noisy sample. This median value is used to replace the original value, completing the filtering process. However, due to the relatively weak ability of IAM filtering to handle Gaussian noise, a significant amount of Gaussian noise tends to remain. A larger region is selected as the window, with the criterion being that within this region, the numerical abrupt changes in the GPR data are not substantial, but they must be noticeably larger than the window size of the IAM filtering. Once the IAM filtering has processed all points within the maximum window, BM3D filtering is used to further process the larger window.

Finally, due to the relatively small abrupt changes in values within a larger window, the variance of data within the window can be used to approximate the variance of Gaussian noise. Subsequently, BM3D filtering is applied using the noise variance to remove Gaussian noise and obtain the final result. The window size of the BM3D filter is 5n × 5n with a maximum of 20 similar blocks.

In the JBI algorithm, both salt-and-pepper noise and Gaussian noise can be effectively filtered out. Meanwhile, the mixed-noise model of Gaussian noise and salt-and-pepper noise is closer to the actual situation, resulting in better filtering effects.

### 2.5. Peak Signal-to-Noise Ratio (PSNR)

To evaluate the accuracy and performance of the noise filtering process, Peak Signal-to-Noise Ratio (PSNR) is used as a standard metric. PSNR is defined using the Mean Squared Error (MSE). For two *m* × *n* images, *I* and *K*, where *I* represents the original clean data and *K* represents the filtered data, their MSE is defined as follows:

(8) $$MSE=\frac{1}{mn}\sum_{i=0}^{m-1}\sum_{j=0}^{n-1}{\left[I\left(i,j\right)-K\left(i,j\right)\right]}^{2}$$

PSNR is defined as follows:

(9) $$PSNR=10\cdot log_{10}\left(\frac{MAX_{I}^{2}}{MSE}\right)=20\cdot log_{10}\left(\frac{MAX_{I}}{\sqrt{MSE}}\right)$$

where MAX is the maximum possible pixel value in the image.

From the above equations, PSNR reflects the quality of noise filtering, where a high PSNR value indicates a better filtering performance and vice versa. The larger the PSNR value, the smaller the difference between the filtered data and the original data, demonstrating the effectiveness of the filtering algorithm.

### 2.6. Finite Difference Time Domain (FDTD)

The Finite Difference Time Domain (FDTD) method is used in this numerical simulation. The basic concept of the FDTD method is to replace the first-order partial derivatives of the field quantities with respect to time and space using central difference quotients. By employing temporal recursion to simulate wave propagation, the method derives the field distribution. The FDTD method directly discretizes the time-domain wave equation without relying on derived equations, thus avoiding limitations on its applicability imposed by certain mathematical models. Its finite difference scheme incorporates medium parameters, allowing for the simulation of various complex structures by assigning appropriate parameters to each grid, which is a significant advantage of the FDTD method. Additionally, since the FDTD method uses a stepping method for computation, it can easily simulate complex time-domain broadband signals and conveniently obtain the time-domain signal waveform at a specific spatial point.

## 3. Results

The proposed JBI algorithm is tested and validated using both synthetic and real GPR data. The data are divided into simple and complex models, both of which are validated using a mixture of Gaussian noise and salt-and-pepper noise as mixed noise in GPR data is close to noises acquired in practice. Mixed-noise scenarios include situations where Gaussian noise dominates, where salt-and-pepper noise dominates, and where the intensities of the two are similar, covering the main possible scenarios faced in practice for the reasonable testing and validation of the proposed JBI algorithm.

### 3.1. Simple Model

**Figure 1:**
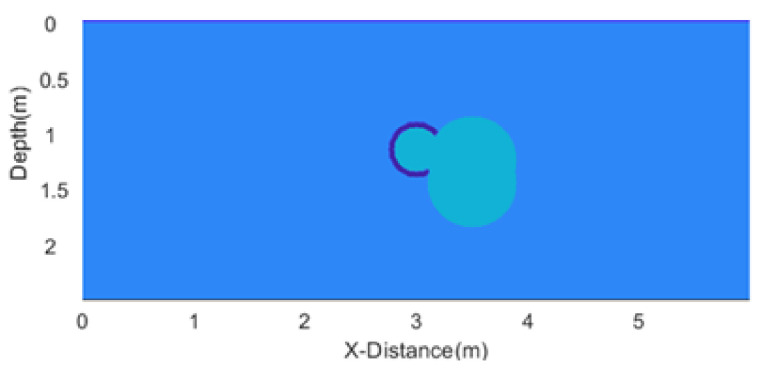
Model of an underground pipe with leaking water.

The first synthetic GPR section is synthesized over a simple model of a leaking underground non-metallic pipe, using the FDTD method for forward modeling as is shown in Figure 1. The model is 6 m × 3 m, and has a 0.01 m grid size in the x direction and 0.01 m grid size in the y direction. The depth to the axis of the pipe is 3 m and the pipe diameter is 0.05 m. The pipe is filled with water and is leaking on its right side. The leakage area is 1.0053 m^2^.

**Figure 2:**
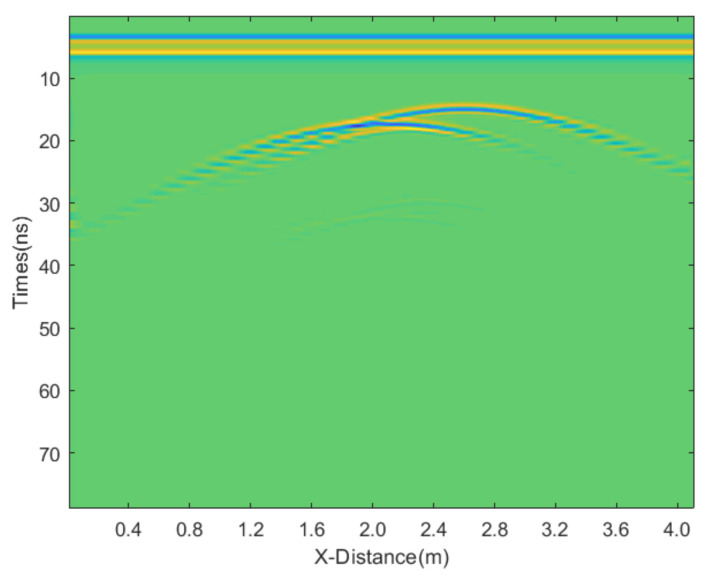
Synthetic GPR section over the model in Figure 1.

The radar antenna is positioned at the air−soil interface. The system uses a central frequency of 500 MHz with a time-sampling interval of 0.0236 ns. The antenna’s transmitting and receiving dipoles are aligned along the *x*-axis. The movement step size of the antenna is 0.4 m, with the transmitting and receiving dipoles in a coaxial configuration. The total scanning distance is 3 m, and the antenna is moved 20 times in total. The time window is set to 80 ns. The relative permittivity and conductivity of different materials are listed in Table 1. The obtained profile is shown in Figure 2.

#### 3.1.1. Equal Intensity Noises

**Figure 3:**
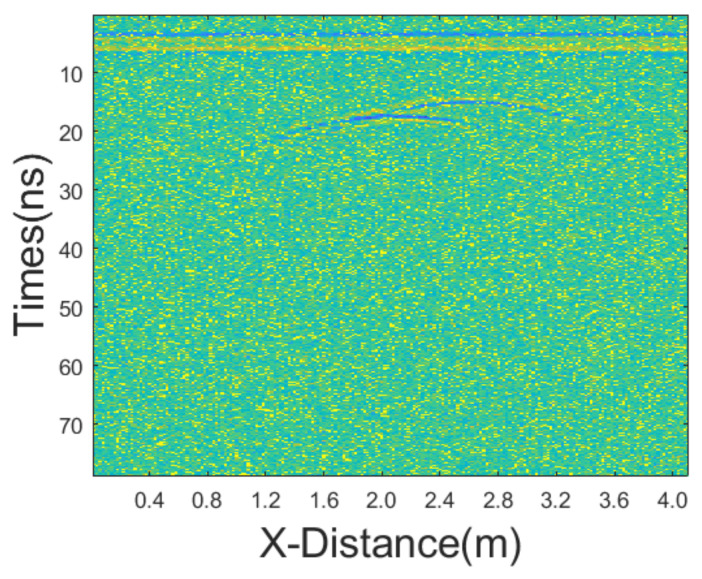
Synthetic GPR section after adding Gaussian noise and salt-and-pepper noise of equal intensities.

In the first testing synthetic GPR section, we added Gaussian noise with a mean of 0 and variance of 25, as well as salt-and-pepper noise with a noise density of 0.1. Figure 3 shows the synthetic GPR section after adding these noises. We employed various methods of denoising, including mean filtering, median filtering, adaptive median (AM) filtering, improved adaptive median (IAM) filtering, BM3D, wavelet transform with hard threshold function (WT-H), wavelet transform with a soft threshold function (WT-S), non-local mean (NLM) filtering, and the proposed JBI filtering. According to Formula (5), the following can be calculated: $windowsizeIAM_{min}=3$. Calculation shows n = 1. So, for the improved adaptive median filtering, the minimum window size is as follows:

$$\left(windowsizeIAM_{min}\cdot n\right)\times \left(windowsizeIAM_{min}\cdot n\right)=3\times 3$$

**Figure 4:**
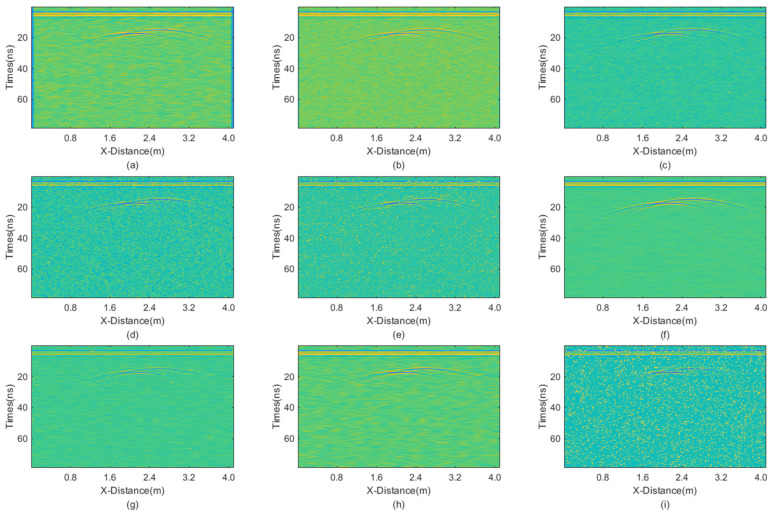
The processed profile section with equal intensities of Gaussian noise and salt-and-pepper noise. (**a**) Mean; (**b**) Median; (**c**) AM; (**d**) IAM; (**e**) BM3D; (**f**) JBI; (**g**) WT-H; (**h**) WT-S; and (**i**) NLM filters.

The incremental step size of the window is 2, and the maximum window size is 7 × 7. The window size of BM3D is 15 × 15. BM3D selects a maximum of 20 similar blocks. The processing results are presented in Figure 4.

From the figure, it is evident that all filtering methods can effectively remove random noise. However, the JBI algorithm undoubtedly demonstrates the best performance. Additionally, the IAM filtering method has basically removed salt-and-pepper noise, leaving only Gaussian noise, while BM3D shows a significant lack of denoising ability for salt-and-pepper noise, which persists in the image. The wavelet transforms with both hard and soft threshold functions also exhibit good filtering effects, but upon close observation, a small amount of noise persists in the image. Finally, the non-local mean filtering method performs the worst.

The calculated PSNR of the processing results obtained using various methods is shown in Table 2. The PSNR results are consistent with the visual observations mentioned above. JBI demonstrates the best performance, followed by wavelet transforms and then other methods. The worst denoising performance is for non-local mean filtering.

These results indicate that when the proportion of salt-and-pepper noise is relatively high, the advantage of improved adaptive median filtering becomes apparent. Meanwhile, when the proportion of Gaussian noise is relatively high, the advantage of BM3D becomes evident. In either case, the proposed joint denoising algorithm exhibits the best performance. In summary, the JBI algorithm yields the best processing effect.

#### 3.1.2. Dominant Gaussian Noise

**Figure 5:**
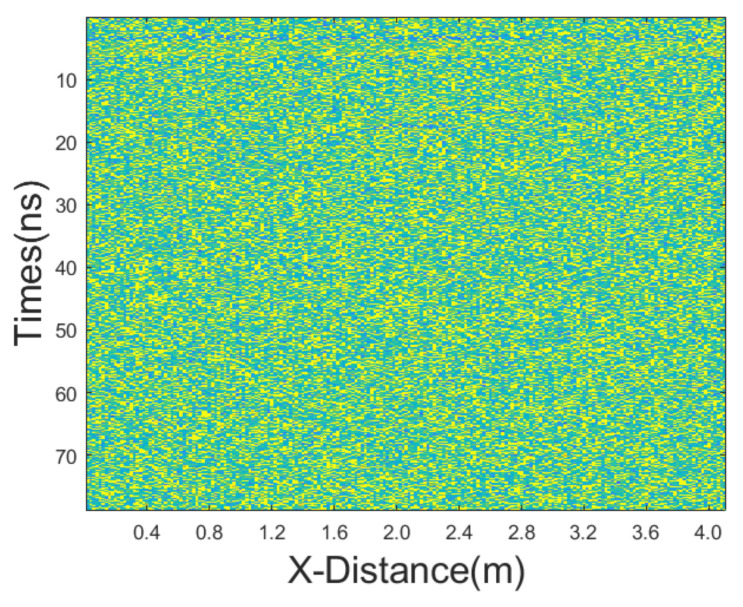
GPR section with dominant Gaussian noise.

We added Gaussian noise with a mean of 0 and a variance of 65, as well as salt-and-pepper noise with a noise density of 0.2. The profile after adding noise is shown in Figure 5. We employed various denoising methods, including mean filtering, median filtering, AM, IAM, BM3D, JBI, WT-H, WT-S, and NLM. According to Formula (5), the following can be calculated: $windowsizeIAM_{min}=5$. The calculation shows n = 1. So, for the improved adaptive median filtering, the minimum window size is:

$$\left(windowsizeIAM_{min}\cdot n\right)\times \left(windowsizeIAM_{min}\cdot n\right)=5\times 5$$

**Figure 6:**
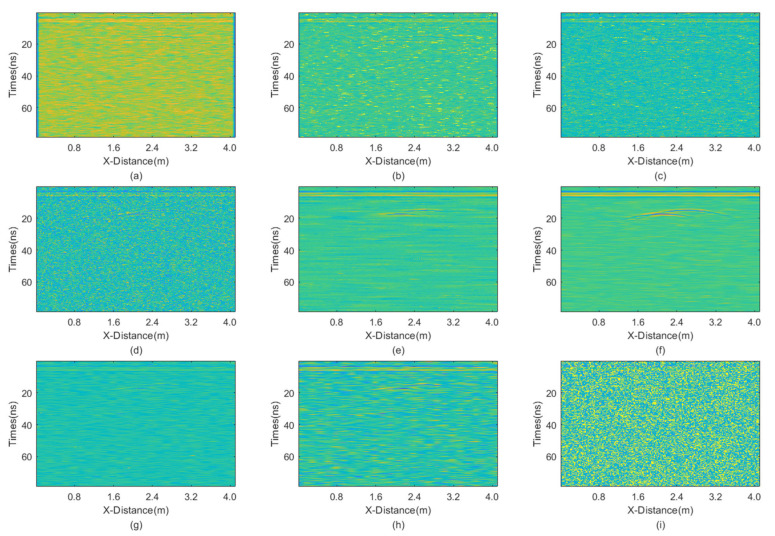
The processed profile section with dominant Gaussian noise. (**a**) Mean; (**b**) Median; (**c**) AM; (**d**) IAM; (**e**) BM3D; (**f**) JBI; (**g**) WT-H; (**h**) WT-S; and (**i**) NLM filters.

The incremental step size of the window is 2, and the maximum window size is 9 × 9. The window size of BM3D is 25 × 25. BM3D selects a maximum of 20 similar blocks. The processing results are presented in Figure 6.

From the figure, it is evident that the processing result of the JBI denoising algorithm is notably superior to other denoising tools. The performance of BM3D is impressive, indicating that its advantage over Gaussian filtering is particularly evident here. Due to the high noise intensity in this case, the processing results of other denoising methods are relatively poor.

The calculated PSNR is presented in Table 3. JBI significantly outperforms other methods in terms of PSNR, demonstrating the best processing effect. BM3D follows closely behind. Other methods yield poorer results, with some even exhibiting negative PSNR values. In summary, the joint denoising algorithm combining improved adaptive median filtering and BM3D achieves the best processing effect.

#### 3.1.3. Dominant Salt-and-Pepper Noise

**Figure 7:**
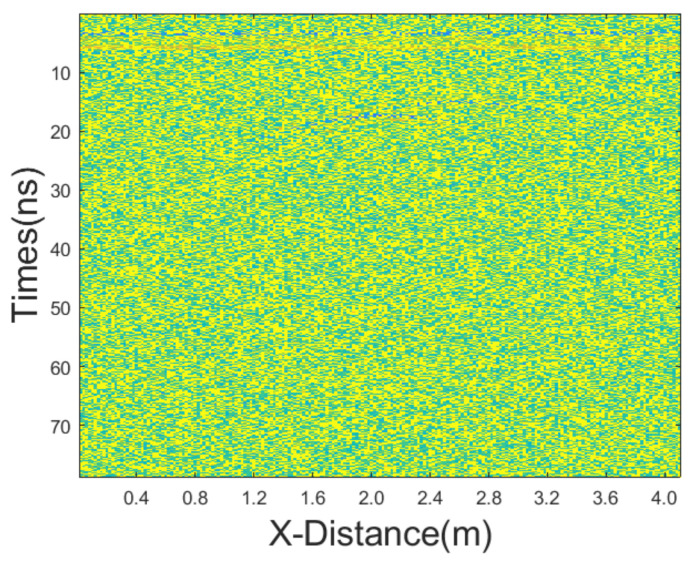
GPR section with dominant salt-and-pepper noise.

**Figure 8:**
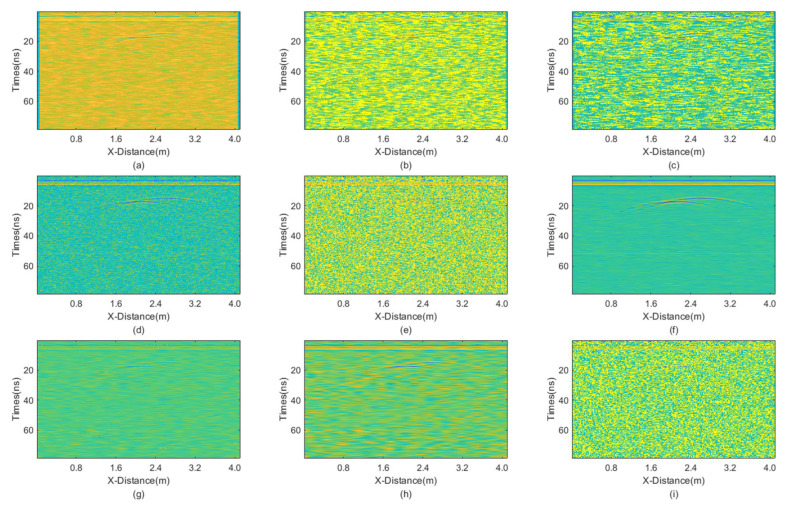
The processed profile section with dominant salt-and-pepper noise. (**a**) Mean; (**b**) Median; (**c**) AM; (**d**) IAM; (**e**) BM3D; (**f**) JBI; (**g**) WT-H; (**h**) WT-S; and (**i**) NLM filters.

In this experiment, we added Gaussian noise with a mean of 0 and a variance of 25, as well as salt-and-pepper noise with a noise density of 0.5. In this case, the mixed noise is primarily salt-and-pepper noise. The GPR section after adding noise is shown in Figure 7. We employed various denoising methods, including mean filtering, median filtering, AM, IAM, BM3D, JBI, WT-H, WT-S, and NLM filters (Figure 8). According to Formula (5), the following can be calculated: $windowsizeIAM_{min}=5$. The calculation shows n = 1. So, for the improved adaptive median filtering, the minimum window size is as follows:

$$\left(windowsizeIAM_{min}\cdot n\right)\times \left(windowsizeIAM_{min}\cdot n\right)=5\times 5$$

The incremental step size of the window is 2, and the maximum window size is 9 × 9. The window size of BM3D is 25 × 25. BM3D selects a maximum of 20 similar blocks.

The figure evidently shows that the JBI algorithm yields the best performance. It effectively removes almost all noise while preserving all image details. Given that synthetic data creation primarily involves salt-and-pepper noise, the improved adaptive median filter also performs well, but it is noticeably inferior compared with the JBI filter. Other denoising algorithms exhibit poor performance, either failing to remove all noise or causing significant loss of useful signals.

The PSNR of various methods shows that the JBI filter still performs the best, as shown in Table 4. The improved adaptive median filtering also performs well, but it is noticeably inferior compared with the joint denoising algorithm. The denoising performance of the other algorithms is relatively poor.

In summary, for simple models, whether the mixed noise is dominated by Gaussian noise, salt-and-pepper noise, or a combination of both, the proposed JBI filter algorithm yields the best denoising results.

### 3.2. Random Equivalent Medium Model

**Figure 9:**
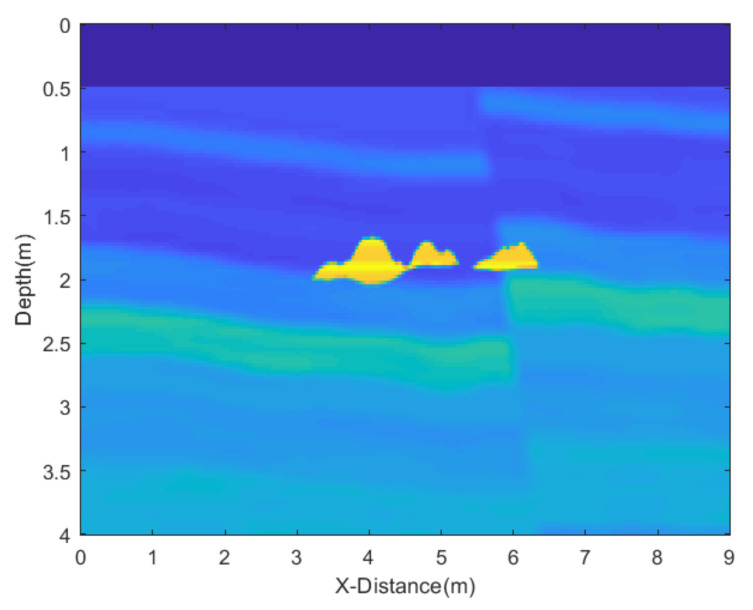
Leaking water model of the underground pipe.

**Figure 10:**
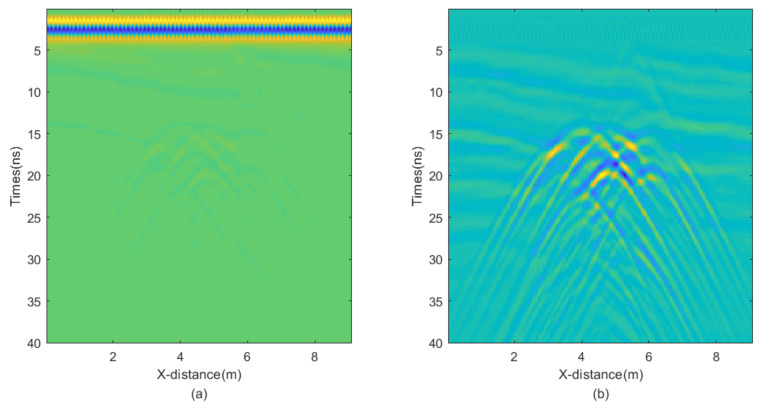
Raw GPR section (**a**) and the same section after removing direct waves and applying automatic gain control (**b**).

In this experiment, we employed a relatively complex random equivalent medium and target to validate our algorithm, with the model diagram illustrated in Figure 9. The uniformly dark blue section represents air, and the ground-penetrating radar moves across the interface between air and the ground. The relative permittivity of the surrounding medium ranges from 2.5 to 6, while the relative permittivity of the target ranges from 9 to 10. The total movement distance is 9 m, and the model depth is 4 m. The finite-difference time-domain (FDTD) algorithm for the numerical simulations is used, with a grid size of 0.025 m × 0.025 m. The central frequency of the antenna is 500 MHz. The resultant GPR section is presented in Figure 10a, while Figure 10b is obtained by removing the direct wave and applying automatic gain control, where the reflected wave information becomes clearly visible. Next, we add noise to the data and apply different denoising algorithms to test the performance of the proposed JBI algorithm.

#### 3.2.1. Equal Intensity Noises

**Figure 11:**
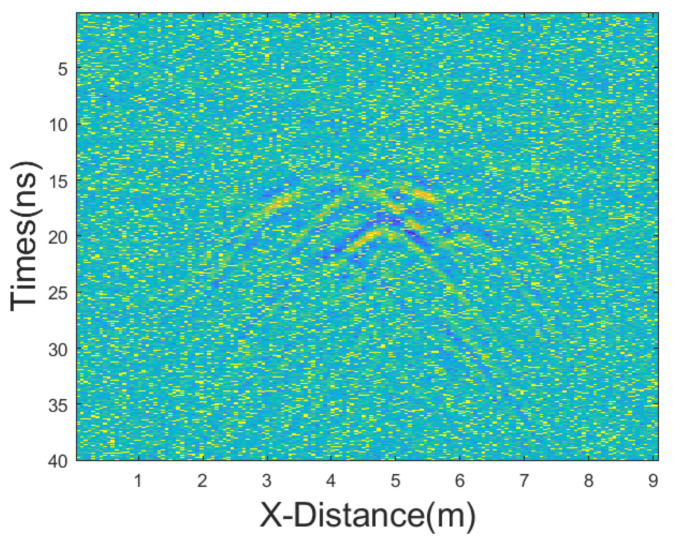
The GPR section from Figure 10b after adding equal intensity noises.

In this experiment, we added mixed noise consisting of Gaussian noise and salt-and-pepper noise. The intensities of Gaussian noise and salt-and-pepper noise in the mixed noise are approximately equal. The mean of Gaussian noise is 0 with a variance of 25. The noise density of salt-and-pepper noise is 0.1. The GPR section after adding the mixed noise is shown in Figure 11. Then, we employed various denoising methods, including mean, median, AM, IAM, BM3D, JBI, WT-H, WT-S, and NLM filters. According to Formula (5), the following can be calculated: $windowsizeIAM_{min}=3$. The calculation shows n = 1. So, for the improved adaptive median filtering, the minimum window size is as follows:

$$\left(windowsizeIAM_{min}\cdot n\right)\times \left(windowsizeIAM_{min}\cdot n\right)=3\times 3$$

**Figure 12:**
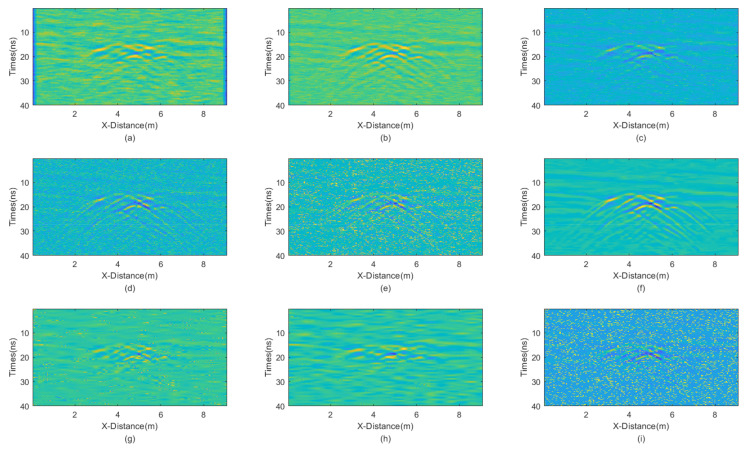
The processed GPR section with equal intensity noises. (**a**) Mean; (**b**) Median; (**c**) AM; (**d**) IAM; (**e**) BM3D; (**f**) JBI; (**g**) WT-H; (**h**) WT-S; and (**i**) NLM filters.

The incremental step size of the window is 2 and the maximum window size is 7 × 7. The window size of BM3D is 15 × 15. BM3D selects a maximum of 20 similar blocks. The processing results are presented in Figure 12.

Careful inspection of Figure 12 shows that JBI exhibits the best denoising effect, with very smooth processing results and prominent effective signals. Other methods do not achieve a satisfactory denoising performance. Mean filtering has always been poor at handling boundaries. Meanwhile, the two methods of wavelet transform, with high and soft threshold functions, demonstrate an insufficient denoising capability and more damage to useful information when dealing with such complex waveform situations. The PNSR of various filtering variance processing results is shown in Table 5.

The PSNR of the JBI algorithm is the highest, indicating its superior filtering performance. The PSNR of other algorithms is significantly lower. This is consistent with the visual inspection of Figure 12.

#### 3.2.2. Dominant Gaussian Noise

**Figure 13:**
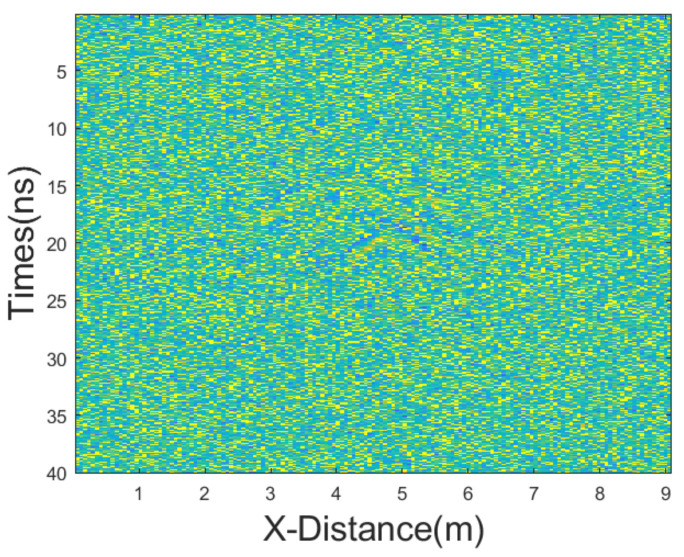
The GPR section displayed in Figure 10b after adding dominant Gaussian noise.

**Figure 14:**
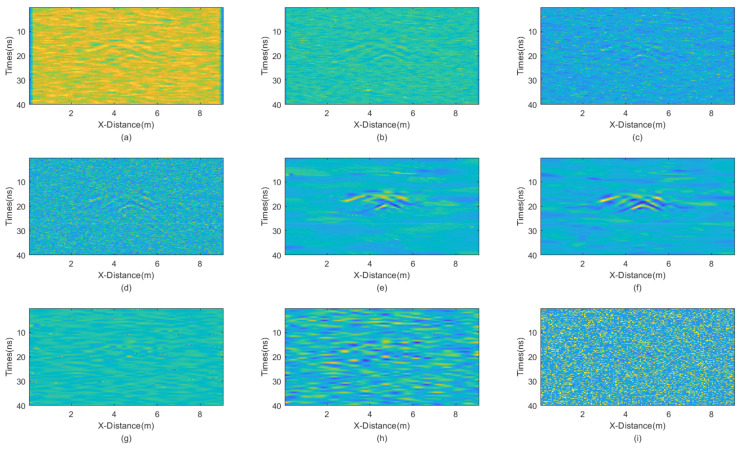
The processed GPR section with dominant Gaussian noise. (**a**) Mean; (**b**) Median; (**c**) AM; (**d**) IAM; (**e**) BM3D; (**f**) JBI; (**g**) WT-H; (**h**) WT-S; and (**i**) NLM filters.

In this case, we added Gaussian noise with a mean of 0 and a variance of 65, as well as salt-and-pepper noise with a noise density of 0.2. The GPR section after adding noise is shown in Figure 13. The results of applying different denoising methods, including mean, median, AM, IAM, BM3D, JBI, WT-H, WT-S, and NLM filters, are shown in Figure 14. According to Formula (5), the following can be calculated: $windowsizeIAM_{min}=5$. The calculation shows n = 1. So, for the improved adaptive median filtering, the minimum window size is as follows:

$$\left(windowsizeIAM_{min}\cdot n\right)\times \left(windowsizeIAM_{min}\cdot n\right)=5\times 5$$

The incremental step size of the window is 2 and the maximum window size is 9 × 9. The window size of BM3D is 25 × 25. BM3D selects a maximum of 20 similar blocks.

From the figure, it is evident that JBI yields the best results, demonstrating a superior protection of useful information. BM3D also performs well in denoising this particular GPR data. However, its damage to useful signals is notably more severe than that caused by the proposed JBI filter. Given the significant overall intensity of mixed noise in this case, only the improved adaptive median filtering among other methods can clearly reveal useful signals. Other methods either fail to filter out noise or cause severe damage to useful signals.

The PNSR calculation results are presented in Table 6. The information reflected by the data is basically consistent with the visual observations. JBI exhibits the best performance and BM3D also performs well, but it significantly damages the useful signals. Other data processing methods yield poorer results.

#### 3.2.3. Dominant Salt-and-Pepper Noise

**Figure 15:**
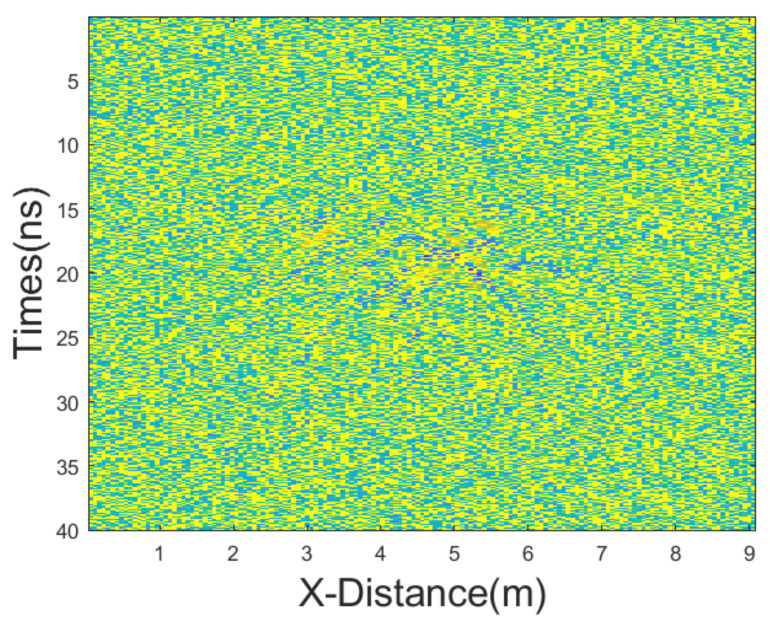
The GPR section displayed in Figure 12b after adding dominant salt-and-pepper noise.

In this last experiment, we added Gaussian noise with a mean of 0 and a variance of 25, and salt-and-pepper noise with a noise density of 0.5. The GPR section after adding these noises is shown in Figure 15. Then, we applied the nine different denoising methods, including the mean, median, AM, IAM, BM3D, JBI, WT-H, WT-S, and NLM filters. According to Formula (5), the following can be calculated: $windowsizeIAM_{min}=5$. Calculation shows n = 1. So, for the improved adaptive median filtering, the minimum window size is:

$$\left(windowsizeIAM_{min}\cdot n\right)\times \left(windowsizeIAM_{min}\cdot n\right)=5\times 5$$

**Figure 16:**
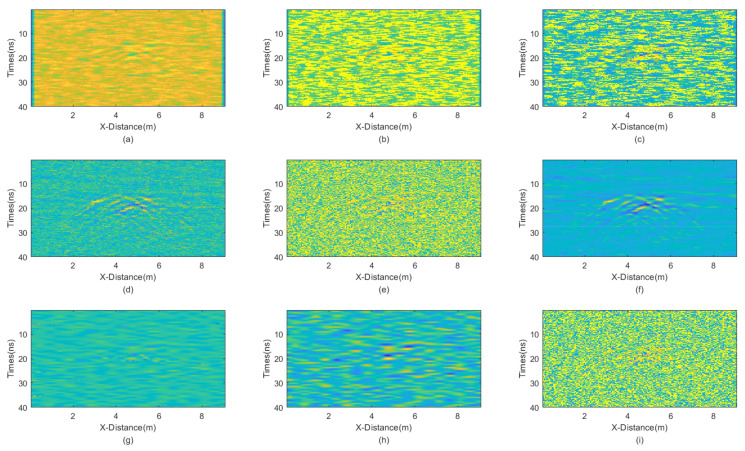
The processed GPR section with dominant salt-and-pepper noise. (**a**) Mean; (**b**) Median; (**c**) AM; (**d**) IAM; (**e**) BM3D; (**f**) JBI; (**g**) WT-H; (**h**) WT-S; and (**i**) NLM filters.

The incremental step size of the window is 2 and the maximum window size is 9 × 9. The window size of BM3D is 25 × 25. BM3D selects a maximum of 20 similar blocks. The denoising results are presented in Figure 16.

From the figure, it is obvious that the JBI denoising algorithm yields the best denoising effect. The improved adaptive median filtering also performs well, but it is notably less effective at handling Gaussian noise. The processing results of other filtering algorithms are unsatisfactory. The calculated PSNR for each processing method, shown in Table 7, are in good alignment with the visual observations.

In conclusion, whether it is a simple or complex model, whether the mixed noise is predominantly Gaussian or salt-and-pepper, or whether the intensities of both are equal, the proposed JBI denoising algorithm yields the best denoising effect compared to the other tested algorithms.

### 3.3. Real Field Data

Synthetic data are always useful for evaluating a new processing tool; however, a successful tool must perform equally as well when applied to real data. Consequently, we tested the proposed JBI algorithm using real field data. The GPR section was 230 m long with a trace interval of 0.02 m. The time-sampling interval was 0.1171875 ns and the antenna’s central frequency was 500 MHz. We employed various denoising methods, including mean, median, AM, IAM, BM3D, JBI, WT-H, WT-S, and NLM filters. According to Formula (5), the following can be calculated: $windowsizeIAM_{min}=3$. The calculation shows n = 1. So, for the improved adaptive median filtering, the minimum window size is:

$$\left(windowsizeIAM_{min}\cdot n\right)\times \left(windowsizeIAM_{min}\cdot n\right)=3\times 3$$

**Figure 17:**
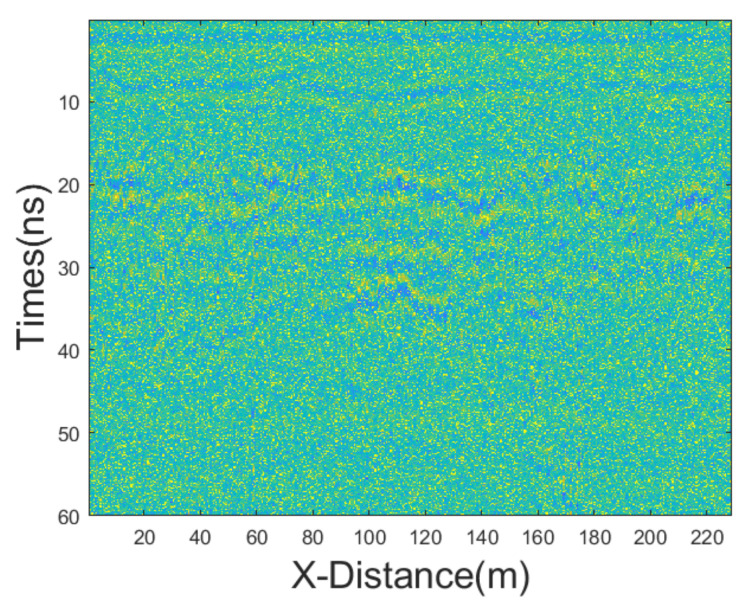
Real GPR section.

**Figure 18:**
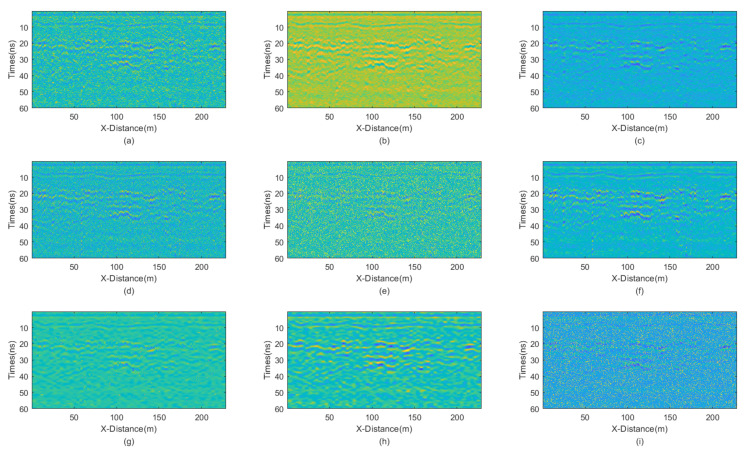
The real GPR section. (**a**) Mean; (**b**) Median; (**c**) AM; (**d**) IAM; (**e**) BM3D; (**f**) JBI; (**g**) WT-H; (**h**) WT-S; and (**i**) NLM filters.

The incremental step size of the window is 2, and the maximum window size is 7 × 7. The window size of BM3D is 15 × 15. BM3D selects a maximum of 20 similar blocks. The original, unprocessed profile is shown in Figure 17, and the processing results are presented in Figure 18.

The figure clearly shows that the filtering performance of the JBI denoising algorithm is the best among the other algorithms. Although the filtering effect of adaptive median filtering is also good, upon close observation, this algorithm still has a lot of noise, albeit relatively minor. The filtering performance of the other algorithms is significantly inferior to that of the proposed JBI algorithm. Either the denoising is not thorough, or the useful information is severely damaged.

It can be confidently stated that the proposed JBI filter outperforms all other filters tested in this research in terms of both attenuating noise and preserving useful signal.

## 4. Discussion

For simple models, the filtering performance of the proposed joint BM3D with an improved adaptive mean (JBI) denoising algorithm yielded the best results in three different scenarios of noise proportions. Whether the mixed noise is dominated by Gaussian noise, salt-and-pepper noise, or both with comparable intensities, the JBI denoising algorithm can largely restore the original signal. This is mainly due to the strong denoising capability of BM3D for Gaussian noise and the improved adaptive median filtering for salt-and-pepper noise. Since the reflected waves of simple models are relatively simple and do not contain too much detailed information, the PNSR of the processing results of the JBI algorithm remain at a high level, proving its excellent filtering performance.

For complex models, specifically the random equivalent medium model, the reflected wave is relatively complex and thus contains more detailed information. Although the filtering performance of the JBI denoising algorithm declines, it still maintains the best filtering performance among the other tested methods. This indicates that our proposed algorithm has broad practical applicability, especially for more complex reflected waves, as it can still preserve image details, effectively filter out random noise, and improve the signal-to-noise ratio.

The noise we introduce to the synthetic data is a mixture of Gaussian noise and salt-and-pepper noise. This approach is more realistic, as real-world data are highly complex and unlikely to be affected by a single type of noise. Gaussian noise is caused by uneven scattering of underground soil particles, while salt-and-pepper noise is due to instrument instability, the presence of large reflection points, or partial data loss. Therefore, Gaussian noise and salt-and-pepper noise are two of the most common types of random noise. Our mixed noise is thus closer to real-world situations. To cover a wider range of applicability, we discuss scenarios where Gaussian noise is dominant, salt-and-pepper noise is dominant, and the noise levels of the two are not significantly different. This covers the most of the main underground situations. Therefore, our numerical simulation is more realistic. This also verifies that the proposed JBI denoising algorithm performs best in denoising under the main situations encountered in practical work. Moreover, when applied to real field GPR data, the proposed JBI denoising algorithm exhibits the best filtering performance among other commonly used denoising tools.

## 5. Conclusions

In this work, we propose and test a joint denoising algorithm, abbreviated as JBI, that combines the capabilities of both BM3D and IAM filters to suppress Gaussian and salt-and-pepper noises. Experiments conducted on synthetic GPR data having different proportions of noises and real field GPR data show that the proposed JBI denoising algorithm exhibits the best filtering performance among the other tested algorithms. The proposed JBI algorithm successfully exploits the denoising advantages of the improved adaptive median filtering against salt-and-pepper noise and the BM3D method against Gaussian noise, thus achieving the best filtering performance and the widest applicability. This significant performance includes good discrimination against different types of noise and a great capability to preserve useful signals of interest.

## Figures and Tables

**Table 1:** Relative permittivity and conductivity of model materials.

Material	Water	Metallic Pipe	Soil
Relative permittivity	80	0	6
Conductivity (S/m)	0.07	1.25 × 10^−7^	4 × 10^−2^

**Table 2:** PNSR of various filtering methods.

Mean	Median	AM	IAM	BM3D	JBI	WT-H	WT-S	NLM
44.498	42.218	55.284	42.783	49.546	73.876	66.752	67.342	14.353

**Table 3:** PNSR of various filtering methods.

Mean	Median	AM	IAM	BM3D	JBI	WT-H	WT-S	NLM
−4.754	−2.053	2.936	1.328	11.365	39.091	−1.350	−7.865	−22.353

**Table 4:** PNSR of various filtering methods.

Mean	Median	AM	IAM	BM3D	JBI	WT-H	WT-S	NLM
−7.097	−6.380	−5.387	10.639	−8.775	41.651	−2.239	−3.378	−23.376

**Table 5:** PNSR of various filtering methods.

Mean	Median	AM	IAM	BM3D	JBI	WT-H	WT-S	NLM
31.273	32.372	55.094	51.370	47.092	67.353	35.373	33.980	8.129

**Table 6:** PNSR of various filtering methods.

Mean	Median	AM	IAM	BM3D	JBI	WT-H	WT-S	NLM
−11.245	−7.251	1.256	8.349	21.267	28.288	−13.236	−15.981	−33.376

**Table 7:** PNSR of various filtering methods.

Mean	Median	AM	IAM	BM3D	JBI	WT-H	WT-S	NLM
−16.465	−14.948	−12.356	15.456	−11.309	33.740	−3.450	−8.307	−24.477

## Data Availability

Data is contained within the article.
